# Specific Reduction of Dopaminergic Fiber Input to Ventrobasal Forebrain Targets in Neonatal Mice Following Prenatal Exposure to Valproate

**DOI:** 10.3390/biomedicines14030590

**Published:** 2026-03-05

**Authors:** Ágota Ádám, Cintia Klaudia Finszter, Gergely Zachar, María Pilar Madrigal, Diego Echevarría, Salvador Martínez, András Csillag

**Affiliations:** 1 Department of Anatomy, Histology and Embryology, Faculty of Medicine, Semmelweis University, 1085 Budapest, Hungary; adam.agota@semmelweis.hu (Á.Á.); finszter.cintia.klaudia@semmelweis.hu (C.K.F.); zachar.gergely@semmelweis.hu (G.Z.); 2 Institute of Neuroscience (UMH-CSIC), University of Miguel Hernández, Campus de Sant Joan, 03550 Sant Joan, Alicante, Spain; mmadrigal@umh.es (M.P.M.); diegoaza@umh.es (D.E.); smartinez@umh.es (S.M.)

**Keywords:** ASD, dopaminergic pathway, mesolimbic system, iDISCO, morphometry, ontogeny, volumetric analysis, neurodevelopmental disorder

## Abstract

**Background/Objectives**: The neuromorphological effects of prenatal administration of valproic acid (VPA) on the dopaminergic system has been studied by our groups for some time. Previously, we found a marked defasciculation of the mesotelencephalic pathway, and a reduction of dopaminergic ventrotegmental output, with diminished dopamine in the nucleus accumbens (NAc) but not in the caudatoputamen (CPu), in VPA exposed P7 mice. Further, we reported a marked decrease in the juxtapositions between tyrosine hydroxylase positive (TH+) axon terminals and calretinin or calbindin containing neurons in the NAc and tuberculum olfactorium (OT). Our aim was to test the existing findings, indicating diminished input of TH+ structures to dopamine recipient forebrain, by another robust and unbiased quantitative approach. **Methods**: Here, the intensity of TH immunolabel was quantified by 3D image analysis of whole-mount, tissue-cleared (by the iDISCO method) brain specimens of P7 mice born to VPA-exposed or control mothers. **Results**: We observed a robust reduction in TH+ immunostaining (expressed as mean voxel intensity within the ROI) in the OT, and a less prominent but significant reduction of this parameter in the NAc, in VPA exposed vs control mice. No such effect was observed in the CPu, indicating that the decrease of DA input affected predominantly the limbic component of dopamine recipient forebrain regions. **Conclusions**: Together with previous observations, the current results seem to converge upon a consistent interpretation, i.e., reduced DAergic fiber input to ventral forebrain regions, following VPA exposure of neonatal mice. Weaker supply of DA at a critical time of embryonic development may result in impaired pattern formation of ventrobasal forebrain regions involved in reward and sociability.

## 1. Introduction

Autism Spectrum Disorder (ASD) represents a highly prevalent neurodevelopmental condition characterized by significant deviations in social conduct. Despite the varied clinical manifestations, all forms of ASD are unified by compromised social adaptation and behavioral deficits, although its underlying etiology remains largely undetermined. Pathology is commonly attributed to two primary categories of risk factors: genetic and environmental/epigenetic. A limited subset of human ASD cases has been definitively traced to established genetic components, representing diagnostic and predictive advancements. Nevertheless, most ASDs are hypothesized to stem from environmental or epigenetic influences during critical periods of embryonic development.

The link between ASD and dopaminergic (DAergic) system dysfunction has long been suggested. This association is supported by evidence ranging from functional impairment [1] and, genomic alterations [2,3] to defective development [4,5] of DAergic structures. Given the ample DAergic input they receive, striatal circuits are frequently implicated in the development of various ASD forms [6]. Furthermore, DAergic dysfunction has been confirmed in animal models of autism [7,8,9,10].

These findings have converged into a prominent “dopamine theory” of human autism [11,12,13,14]. Unlike many other proponents of this hypothesis, we envisage DA as a promoter of forebrain development. This role was initially proposed for striatal development by Voorn et al. [15] and supported by ex vivo co-culture studies on target neurons [16]. By extrapolation, DAergic input likely affects pattern formation in the ventrobasal forebrain, a key component of the social brain network. Our working hypothesis is that weak or delayed DAergic input—one that misses the critical developmental time point—can result in weaker adaptation to social signals, ultimately manifesting as ASD.

Gestational exposure of laboratory rodents to valproic acid (VPA) in a narrow time window of development is an established experimental model for investigating the phenotypic deficits of sociability associated with ASD. Administration of VPA, a compound used as an anticonvulsant, antiepileptic, and mood stabilizer [17], brings about characteristic deficits in the social behavior of offspring [18,19,20,21,22,23]; for comprehensive reviews, see [21,24,25]. VPA exposure as a relevant model is not confined to mammals, it also works in fish [26,27] and birds [28,29,30,31,32]. The VPA model provides a pertinent framework for exploring the role of epigenetic/environmental factors in the etiology of ASD [33].

The likely mechanisms by which VPA treatment induces autism-like symptoms are the alteration of Wnt-1 expression [34,35] and, predominantly, the inhibition of histone deacetylase, leading to modified gene transcription [23,36,37] during a critical phase of embryonic development. Previous studies indicate that the optimal time window for VPA administration to elicit ASD-like behaviors without major teratogenic effects, is embryonic day E12–15 [37]. This specific prenatal period is concurrent with two crucial neurodevelopmental events: the early differentiation of midbrain DAergic neurons [38,39] and the outgrowth and rostral migration of DAergic axons targeting the ventrobasal forebrain [40].

Regarding the VPA model, altered distribution of mesencephalic DAergic neurons has been described in domestic chicks [41]. Our research groups demonstrated specific morphological and neurochemical alterations of rodent DAergic system (defasciculation of mesotelencephalic pathway, shift in the distribution of midbrain DA neurons, reduced DA le vel in nucleus accumbens, NAc) [42]. This finding aligns with an fMRI investigation reporting perturbed connectivity of the mesolimbic reward pathway in human subjects [43]. We suggested that reduced entry of DA fiber at a critical stage of ontogeny could result in impaired patterning within the dopaminoceptive target regions.

Further, our group reported selective reduction of the TH protein/synaptophysin ratio in the NAc, but not in CPu, as detected on western blots from VPA exposed vs control mice [44]. In a subsequent study, employing double immunostaining of brain sections, confocal laser microscopy and 3D image analysis, we found a marked decrease in the density of the juxtapositions between TH+ axon terminals and CR+ neurons (in NAc), as well as those between TH+ axons and CB+ neurons (in OT), in VPA exposed animals [45]. These findings provided further pieces of evidence for a diminished input of TH+ axons, reducing the likelihood of forming synaptic contacts in the target areas. Our data obtained by different independent methods seem to converge upon a consistent interpretation, i.e., the weakening of DAergic input to ventral forebrain regions, at least in neonatal mice born to VPA exposed mothers.

In contrast to our established findings [42,44,45], a separate investigation reported no observed differences in the abundance of DAergic neurons within the SN or the VTA, nor in the expression of TH in the CPu or NAc of mice following prenatal VPA exposure [46]. However, the authors [46] observed an upregulation of DA receptors, suggesting, in retrospect, previous deprivation of the ligand (DA). Similarly, Schiavi et al. [47] reported an upregulation of D2 receptors (at P35–40) and then D1 receptors (at P90–95), in the NAc of VPA-exposed rat offspring. Both sets of findings can be interpreted as compensatory receptor upregulation stemming from an earlier depletion of the ligand (DA), which is consistent with the DA reduction we detected in P7 mice.

Crucially, most of these observations [46,47] were made in juvenile post-weaning (P21) or adult rodents. At these later stages, the DAergic organizational processes–which begin in the late embryonic period and undergo protracted postnatal development [48]–are largely considered consolidated. By contrast, in our reports, P7 animals were used consistently for VPA-control comparisons [42,44,45], aiming to investigate the developmental processes occurring at the early phase of pattern formation. This time window precedes the cessation of DAergic fiber ingrowth and the final stages of synapse formation between DAergic afferents and their recipient structural elements. This approach allows for the examination of initial defects rather than their long-term, consolidated consequences.

Apart from other differences in methodology, it is this age difference that might well be accounted for the discrepant findings. In our hand, one advantage of using 7-day-old animals was that, at this stage, the ingrowth of DA axons to target regions is still in progress, enabling regulation of patterning. Another bonus of working with animals of this age was a low myelin content, compared to older animals, which proved critical in reducing background noise, when using the iDISCO technique.

Even if age difference is likely to account for the contradictory findings, we decided to promote clarification of the problem by yet another approach: direct measurement of staining intensity of TH+ elements in whole-mount brain specimens from VPA exposed or vehicle-exposed P7 mice.

The age of mice (P7) was chosen for comparability with our previous observations [42,45]. A combination of the iDISCO tissue clearing method, optimization of up-to-date image analysis technique (using the Imaris software, Bitplane AG, Switzerland, version 9.9.1.), together with normalization of signal intensity made it possible to fully exploit the power of unbiased quantitative volumetric analysis in 3D tissue samples.

According to our working hypothesis, reduced dopaminergic input to ventrobasal forebrain target regions (primarily the olfactory tubercle and nucleus accumbens) at a decisive time point of ontogeny would likely perturb the development and consolidation of neural and synaptic architecture in these regions, specifically involved in motivation and reward. Given certain discrepant observations in parallel research, however, it is essential that the notion of a distinct drop of dopaminergic axons entering these forebrain regions, following VPA exposure, be based on unquestionable experimental evidence, at least at one given age (P7) of animals. Only by building upon this firm basis, can we move on to investigate further morphological and functional sequels, including potential restitution, in older age groups.

Critical comparison of our results with other reports has drawn attention to the importance of specific anatomical localization. At variance with several previous studies, using ‘striatal’ samples, not distinguishing ventral (NAc) from dorsal (CPu) subdivisions (e.g., for biochemical analysis), we consistently endeavored to analyze OT, NAc and CPu with their respective DAergic pathways separately, since the focus and extent of responses may vary substantially even in nearby regions [45].

## 2. Materials and Methods

### 2.1. Experimental Animals

The experimental animals of the C57BL/6J strain were purchased from the Laboratory Animal Breeding Facility of the Institute of Oncology, Budapest. The animals were housed at 24 °C under a 12:12 h light–dark cycle, with food and water ad libitum. All housing conditions fully complied with internationally accepted standards for the care and use of laboratory animals.

Following mating, females were examined daily for the presence of vaginal plugs. The first midday when a plug was detected was designated as embryonic day 0.5 (E0.5), and the day of birth was recorded as postnatal day 0 (P0).

On embryonic day 13.5 (E13.5), half of the pregnant females (7 animals) received an intraperitoneal injection of 500 mg/kg VPA (dissolved in physiological saline). The remaining pregnant females (7 animals) received physiological saline injections and served as controls. Seven-day-old (P7) pups born to VPA exposed and control mothers were randomly selected for whole-mount (iDISCO) immunohistochemistry, tissue clearing and light-sheet microscopy (7 animals per treatment group).

For demonstrative purposes only (not included in statistical analyses), another 10 randomly selected mouse pups (5 animals from the VPA-treated and control group each) were used to obtain representative coronal sections for immunohistochemistry.

In both experiments, the mice were terminally anesthetized with ketamine (50 mg/mL) and xylazine (20 mg/mL), injected i.p.in total volume of 0.1 mL. The animals were perfused through the left ventricle of the heart with a solution of 4% paraformaldehyde in PBS, at 4 °C. The brains were removed from skull and postfixed in the same solution until further processing.

### 2.2. iDISCO, Light Sheet Microscope, Image Processing

#### 2.2.1. Whole-Mount Immunocytochemistry and Tissue Clearing (iDISCO)

The protocol essentially followed the description by Renier et al. [49], with minor modifications. The fixed whole brains were washed for 48 h in PBS under continuous agitation, followed by sequential dehydration in 25%, 50%, 75%, and 100% methanol in PBS (90 min per step). As a next step, the samples were bleached overnight in 3% H_2_O_2_ in 100% methanol, and then rehydrated in 100%, 75%, 50%, and 25% methanol in PBS (90 min each).

For permeabilization and blocking, the pretreated brains were incubated in PBS-GT (PBS containing 0.2% gelatin, 0.5% Triton X-100) and 0.1% sodium azide for 3 days at room temperature under agitation. Thereafter, the samples were incubated with the primary anti-TH antibody (rabbit polyclonal IgG, Merck Millipore, catalog #AB152, Burlington, MA 01803, USA) diluted 1:500 in PBS-GT for 7–10 days at 37 °C in a humidified incubator, VWr Incu-Line IL10, VWR International bvba, Leuven, Belgium.

After incubation with the primary antibody, the brains were washed in PBS-GT for 24 h (with 7–8 changes of solution) and incubated with a 1:500 solution of secondary antibody (goat anti-rabbit Cy5; emission 649–670 nm, Invitrogen, Thermo Fisher Scientific, Waltham, MA 02451, USA, catalog #A10523) in PBS-GT for 24 h at 37 °C, filtered through a sterile cellulose acetate syringe filter (0.2 μm), Merck Millipore, Burlington, MA 01803, USA.

Following secondary antibody incubation, brains were washed again in PBS-GT for 24 h (7–8 solution changes), then dehydrated in methanol (50% in PBS, 80% in distilled water, and 100% twice, 1.5 h per step) and cleared overnight in a 1:2 mixture of methanol/dichloromethane (DCM; Sigma-Aldrich, St. Louis, MO 63103, USA, catalog #270997). From this step onward, the vials containing samples were wrapped in aluminum foil for light protection. For final clearing, the brains were incubated in 100% DCM for 2 h at room temperature until they sank to the bottom of the vial, and were transferred to 100% dibenzyl ether (DBE; Sigma-Aldrich, catalog #108014) for at least 30 min, changing solution twice before imaging.

#### 2.2.2. Light Sheet Microscopy:

The specimens were scanned with the help of a light sheet microscope (Ultramicroscope, LaVision BioTec GmbH, 33617 Bielefeld, Germany) equipped with an Olympus MVX10 objective lens system. The brains were fixed onto a specimen holder enabling standard orientation and immersed for imaging in 99% ethyl cinnamate (Sigma-Aldrich, catalog #112372). For image acquisition ImSpector Pro software v144 (LaVision BioTec GmbH, 33617 Bielefeld, Germany) was used, with Z-step set at 7 μm under 1.25× magnification.

#### 2.2.3. Image Processing:

3D reconstruction images of whole brain were rendered from Z-stacks of light sheet scans, with an image analysis system (IMARIS version 9.9.1). For quantitative morphometric analyses, regions of interest (ROIs) were selected according to the Allen Mouse Brain Atlas (©2021 Allen Institute for Brain Science. Mouse Brain Connectivity). The ROIs were delineated manually on the 3D reconstruction images appearing on the screen. The precise selection of ROIs was further assisted by the rotation of 3D images (Supplementary Materials: Video S1, Video S2).

During the analysis, we examined the NAc, OT and CPu for intensity of TH immunolabeling, using the IMARIS image analysis software package (version 9.9.1). Further details of regional selection and volumetric analysis are explained in the Section 3.

### 2.3. Immunocytochemistry in Brain Sections, Fluorescence Microscope and Image Processing

The brains were immersed in 30% sucrose and cut into 30 µm coronal slices using a Leica SM2000R sliding microtome with a freezing stage (Leica Biosystems Nussloch GmbH, D-69226 Nussloch, Germany). Sections were stored in 0.01% sodium azide-containing PBS at 4 °C until further processing. To avoid batch effects, the sections from control and VPA-treated animals were processed in parallel using the same batches of reagents under identical conditions.

Sections were rinsed in PBS (3 × 10 min) and blocked in 5% normal horse serum (NHS) for 30 min. Then the slices were incubated overnight with the primary antiserum (in PBS containing 1% NHS and 0.3% Triton X-100). For primary antibody, anti-tyrosine hydroxylase (rabbit, 1:1000; LOT 3870479, Merck Millipore, Burlington, MA 01803, USA) was used.

After washing in PBS (3 × 10 min), sections were incubated for 1 h with the corresponding secondary antibody (donkey anti-rabbit Alexa Fluor 488), followed by an additional washing step (3 × 10 min in PBS). The sections were mounted under coverslips onto glass slides with a 1:1 glycerol/PBS mounting medium.

For illustrative purposes, specimens were examined and photographed under a LSM 780 confocal laser microscope (Zeiss, Jena, Germany) at 10×, 20×, or 40× magnification. In this case as well, image processing was performed using the Imaris software package version 9.9.1.

### 2.4. Statistical Methods

Statistical analysis was performed on individual hemispheric data (individuals were included into the models as random factor), using the SPSS program, version 27.0.1.0., IBM Corp. Armonk, NY, USA.

To eliminate background staining and normalize the quantitative results, the mean voxel intensity data from OT, NAc and CPu were divided by those obtained from the SSC region, as this area did not exhibit treatment- or side-induced changes. VPA and control groups were compared using generalized mixed linear models with VPA treatment as a fixed effect and hemispheres as a within-subject factor separately for each brain region. Notably, the VPA treatment did not affect laterality (there was no significant interaction between side and treatment).

## 3. Results

**Figure 1:**
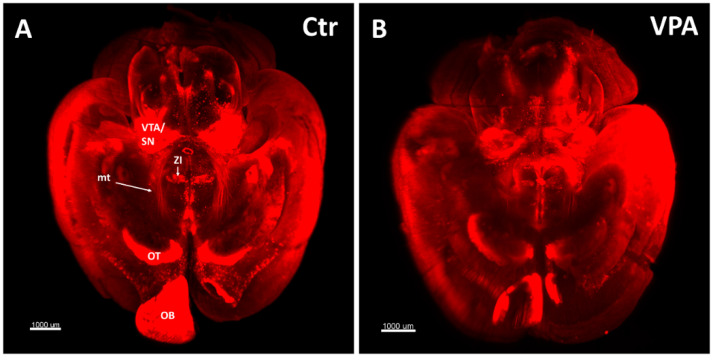
Whole-mount P7 mouse brains were immunostained for TH using the iDISCO method for combined immunolabeling and tissue clearing. (**A**) Control (Ctr) mouse born to a vehicle-treated dam. (**B**) VPA-treated (VPA) mouse born to a sodium valproate-exposed mother. In the VPA specimen, a marked reduction in the density of the mesotelencephalic dopaminergic pathway (mt), as well as in the labeling intensity of the olfactory tubercle (OT) is observed. Notably, the fine scattered surface labeling of cortex represents perivascular sympathetic axonal varicosities of pia. This phenomenon has no effect on the analysis of deeper-lying brain tissue. Scale bar: 1000 µm. Abbreviations: OB, olfactory bulb; ZI, zona incerta; VTA/SN: ventral tegmental area/substantia nigra.

When comparing whole-mount brain specimens, immunostained against TH, from control and VPA exposed mice, the olfactory tubercle (OT) showed a prominently weaker staining intensity in the VPA-exposed group (Figure 1). Lacking DAergic perikarya here, this observation indicated a diminished DAergic axonal input. The previously described [41] defasciculation of the mesotelencephalic tract (mt), following VPA treatment, could also be observed in these specimens.

**Figure 2:**
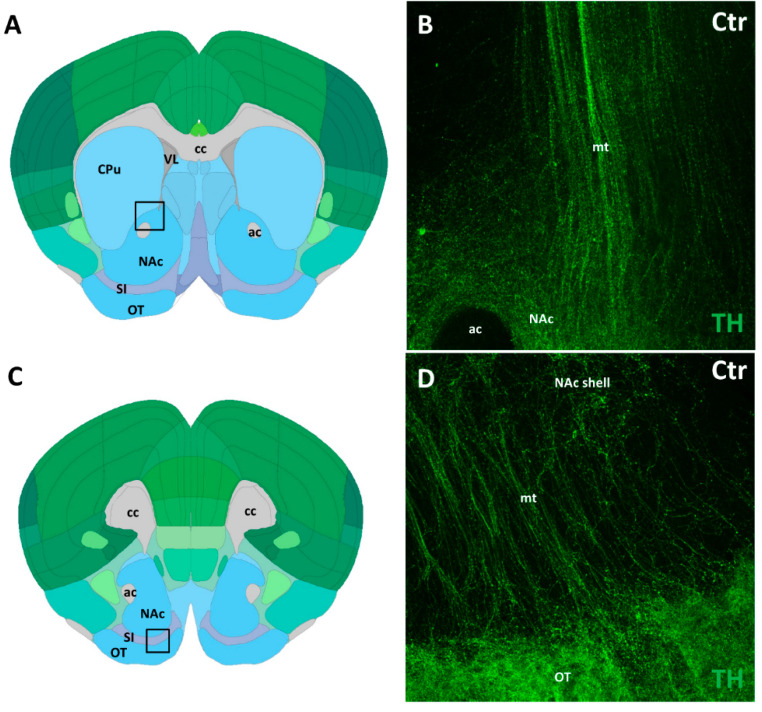
Diagrams (**A**,**C**) indicate the placement of sampling frames within the regions of interest. In (**A**), the sampling frame corresponds to a representative confocal image (**B**) of the mesotelencephalic dopaminergic pathway (mt), immunolabeled for TH, terminating at the medial aspect of the nucleus accumbens (NAc). In (**C**), the sampling frame corresponds to a representative confocal image (**D**) of the TH-immunolabeled mesotelencephalic dopaminergic pathway, traversing the substantia innominata (SI), and entering the medial aspect of the olfactory tubercle (OT). Abbreviations: ac, anterior commissure; cc, corpus callosum; CPu; caudatoputamen; VL: lateral ventricle.

In order to quantify the visual impression of diminished TH in the OT, and to search for similar changes in other, deep-lying DA-recipient forebrain centers (NAc and CPu), the TH+ labeling intensity was systematically analyzed using Imaris 9.9.1. software. For optimization of sampling, the following conditions had to be met. First, we traced the precise course of mt with respect to the non-limbic (CPu) and the limbic (NAc and OT) striatal regions (Figure 2) in confocal laser microscope images of TH-immunostained tissue slices (from control animals), using the Allen Brain Atlas for reference. The mt pathway approaches the NAc and OT from a dorsomedial direction, one part terminating in the medial aspect of NAc, and another part entering the mediodorsal aspect of OT, after traversing the substantia innominata (SI). This topography was taken into consideration at the selection of ROI in whole-mount specimens. The NAc was included in all specimens in its entirety, however, due to its superficial position, the OT was more exposed to surface damage or unspecific labeling. We, therefore, systematically selected a ROI from the dorsomedial aspect of OT (representing layer 3), which was present in all specimens. In the case of NAc, the ROI was generated by 3-D rendering of the entire nucleus (excluding the space occupied by the anterior commissure), whereas the ROIs of CPu and those of SSC (see below) were delineated as hexagonal tissue plugs according to consistent coordinates within the nuclei.

**Figure 3:**
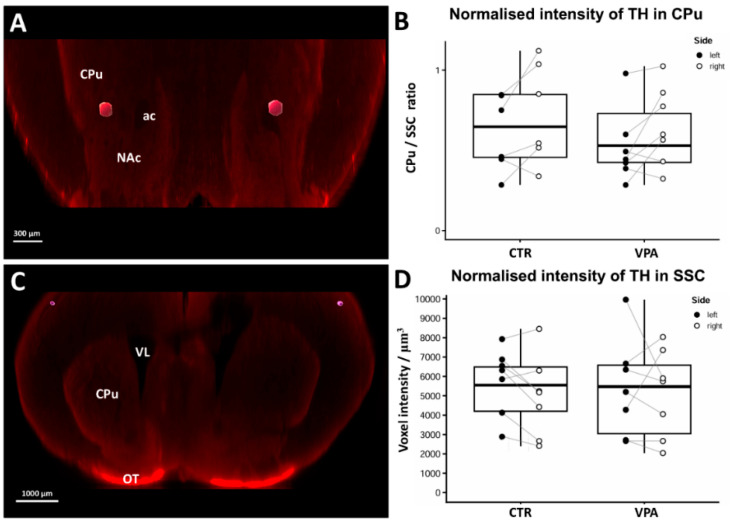
(**A**,**C**) Representative coronal sectional images from whole-mount specimen of a P7 mouse brain (control), immunostained against TH, obtained using the iDISCO method, following user-defined segmentation and volume rendering of ROI by Imaris. The hexagonal profiles (representing rostro-caudally oriented tissue columns) are located in the central portion of CPu (**A**) or in SSC (**C**), in which TH signal intensity was quantified. Scale bars: 300 µm (**A**) and 1000 µm (**C**). (**B**) Diagram depicting the change in TH signal intensity in the CPu in response to VPA treatment, normalized by ipsilateral SSC values. Analyses were performed on P7 mice (control group: *n* = 11 hemispheres, representing 6 animals; VPA-treated group: *n* = 14 hemispheres, representing 7 animals). The box plots depict the distribution of datapoints, lateralized by hemispheres. Effect of treatment: *p* = 0.504 (n.s.), Effect of lateralization: *p* = 0.006. (**D**) Diagram depicting the change in TH signal intensity in the SSC, following VPA treatment. Comparison was performed on P7 mice (CTR: *n* = 14 hemispheres, representing 7 animals; VPA-treated group: *n* = 14 hemispheres, representing 7 animals). The box plots depict the distribution of datapoints, lateralized by hemispheres. Effect of treatment: *p* = 0.898 (n.s.), Effect of lateralization: *p* = 0.231 (n.s.). Abbreviations: ac, anterior commissure; CPu; caudatoputamen; NAc, nucleus accumbens; OT, olfactory tubercle; SSC: somatosensory cortex; VL, lateral ventricle.

**Figure 4:**
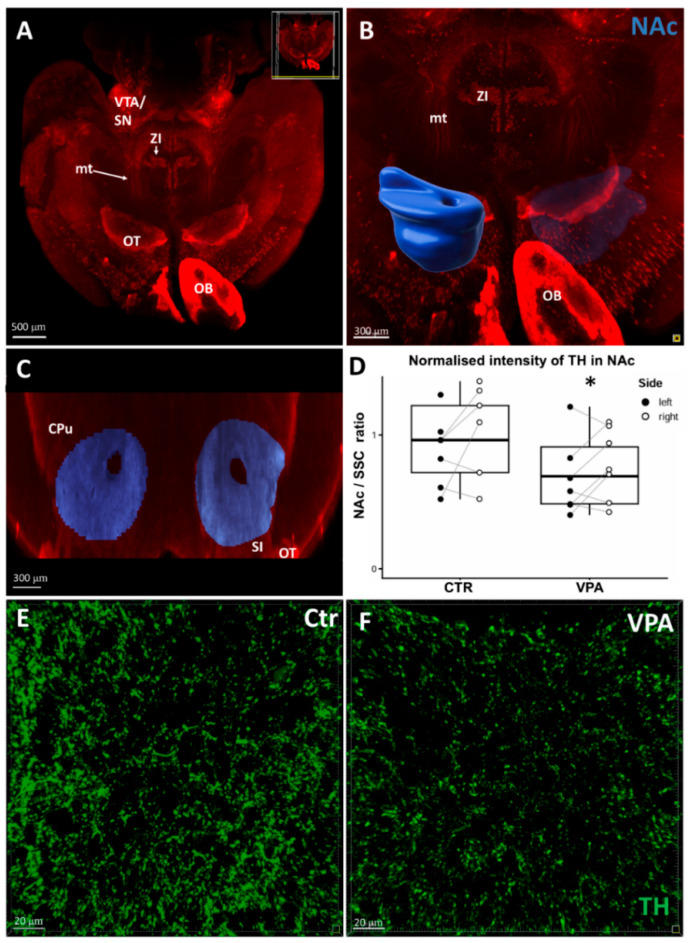
(**A**) Reconstructed 3D light-sheet microscopic image of a typical whole-mount specimen of P7 mouse brain (control) immunostained against TH, obtained using the iDISCO method of combined immunolabeling and tissue clearing. (**B**) Representative 3D image of the same brain following user-defined segmentation and volume rendering of ROI, corresponding to the NAc (shell and core, coded blue). Scale bar: 500 μm. (**C**) Representative coronal sectional image from the region delineated in panel (**B**). Scale bar: 300 μm. (**D**) Diagram showing the change of TH signal intensity within the ROI (NAc, delineated in (**A**–**C**)), normalized by the value of ipsilateral SSC, in response to VPA treatment. The comparison was performed on P7 mice (Control group, CTR: *n* = 13 hemispheres, representing 7 animals; VPA-treated group: *n* = 14 hemispheres, representing 7 animals). The box plots depict the distribution of datapoints, lateralized by hemispheres. Effect of treatment: * *p* = 0.039, effect of lateralization: *p* = 0.030. (**E**,**F**) Representative confocal laser scanning coronal images, acquired at 40× magnification, illustrating changes in the density and distribution of TH-immunolabeled axonal profiles in control (**E**) and VPA-exposed (**F**) mice. Scale bar: 20 um. Abbreviations: ac, anterior commissure; CPu; caudatoputamen; mt, mesotelencephalic pathway; OB, olfactory bulb; OT, olfactory tubercle; SI, substantia innominata; SN, substantia nigra; VTA, ventral tegmental area; ZI, zona incerta.

Another prerequisite for quantification was finding a method for normalization, to eliminate differences in overall staining intensity, occurring during whole-mount immunohistochemistry and tissue clearing. To this end, we selected the somatosensory cortex (SSC) as reference standard, a brain region that fell outside of reward-related basal ganglia, with measurable DAergic axonal input but no TH+ cell bodies. In our opinion, a region with high content of perikaryal TH (such as SN or ZI) would not have served as appropriate standard of staining intensity for those regions, in which the TH signal arose from a far more dispersed (axonal) tissue source. Based upon this method, in the CPu (Figure 3A) the normalized voxel intensity values did not differ significantly between control and VPA-exposed specimens (Figure 3B). Importantly, in the SSC (Figure 3C) the mean voxel intensity values showed no difference between the treatment groups either (Figure 3D), corroborating that this brain region could indeed be used as a stable basis for normalization. Nor was any hemispheric difference observed in the SSC, unlike in CPu (Figure 3B) and in NAc (Figure 4D). Since treatment had no effect on these, hemispheric differences will not be discussed later in the article.

Next, Figure 4 shows the result of volume rendering of NAc (B) projected onto the original light-sheet microscopic image (A), along with a frontal sectional reconstruction image of NAc (C). The mean voxel intensity measured in the ROI of NAc proved to be significantly lower in the specimens from the VPA-exposed group (D). The bottom pair of images demonstrates the density of TH+ axons and varicosities in NAc, qualitatively comparing confocal microscopic images from section-immunostained control (E) and VPA exposed (F) brain specimens. The latter images are shown for demonstration purposes, not for morphometry, since the resolution of whole-mount specimens is not sufficient to discern individual axons.

**Figure 5:**
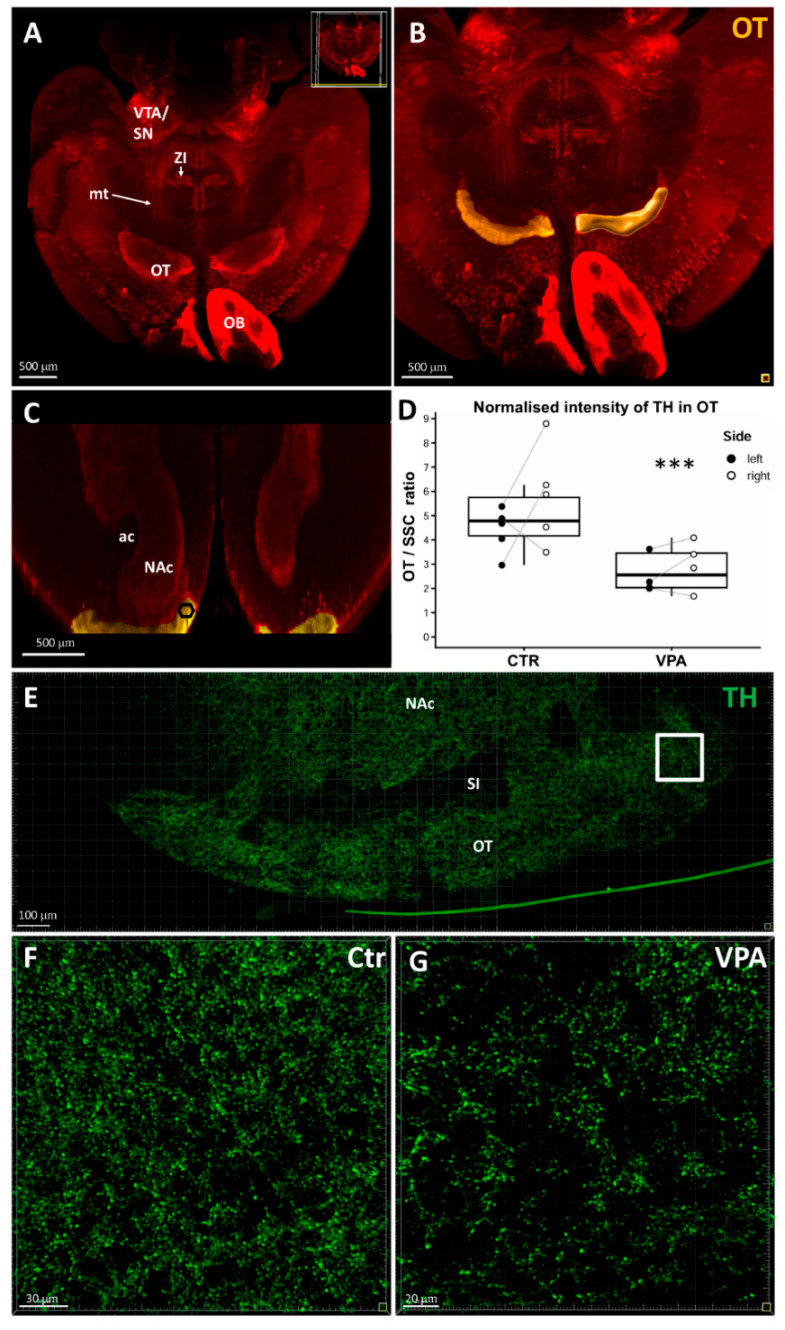
(**A**) Reconstructed 3D light-sheet microscopic image of a typical whole-mount specimen of P7 mouse brain (control) immunostained against TH, obtained using the iDISCO method of combined immunolabeling and tissue clearing. (**B**) Representative 3D image of the same brain following user-defined segmentation and volume rendering of OT (coded yellow). Within this yellow field, a hexagonal ROI was defined in the dorsomedial aspect of OT, in which the intensity of TH signal was measured. Scale bar: 500 μm. (**C**) Representative coronal sectional image to show the position of ROI in OT. Scale bar: 300 μm. (**D**) Diagram showing the change of TH signal intensity within the ROI (dorsomedial aspect of OT, delineated in (**A**–**C**)), normalized by the value of ipsilateral SSC, in response to VPA treatment. The comparison was performed on P7 mice (Control group, CTR: *n* = 10 hemispheres, representing 7 animals; VPA-treated group: *n* = 8 hemispheres, representing 5 animals). The box plots depict the distribution of datapoints, lateralized by hemispheres. Effect of treatment: *** *p* = 0.001, effect of lateralization: *p* = 0.073 (n.s.). (**E**) Overview confocal image of the entire OT, with a square indicating the sampling site (in whole-mount specimens) for the measurement of TH signal intensity in the medial portion of the OT. (**F**,**G**) Representative confocal laser scanning coronal images, acquired at 40× magnification, illustrating changes in the density and distribution of TH-immunolabeled axonal profiles in control (**F**) and VPA-exposed (**G**) mice. Scale bar: 30 µm (**F**), 20 µm (**G**). Abbreviations: ac, anterior commissure; CPu; caudatoputamen; mt, mesotelencephalic pathway; NAc, nucleus accumbens; OB, olfactory bulb; SI, substantia innominata; SN, substantia nigra; VTA, ventral tegmental area; ZI, zona incerta.

In the next photographic plate (Figure 5), similar to Figure 4, the original light-sheet microscopic image (A) is followed by the volume rendering image of OT (B). However, for reasons explained above (Figure 2), the latter was not used for voxel intensity measurement, instead, the ROI selected lay in the dorsomedial aspect of OT, as demonstrated by the hexagon in the frontal sectional image (C). By using this approach, the mean voxel intensity of OT was found to be significantly lower in the specimens from the VPA-exposed group (D), and this difference was even more prominent than in the case of NAc. Figure 4E shows the precise position of measuring frame in an enlarged confocal microscopic image of OT, whereas the comparative density of TH+ axons and varicosities in OT is demonstrated qualitatively in confocal microscopic images from section-immunostained control (F) and VPA-exposed (G) brain specimens.

Overall, the findings are consistent with a robust reduction in TH+ immunostaining (expressed as mean voxel intensity within the ROI) in the OT, and a less prominent but significant reduction of this parameter in the NAc, in whole-mount brain specimens of P7 mice prenatally exposed to VPA, as compared to control mice. No such effect was observed in the CPu, indicating that the decrease of DA input was not uniform, rather it affected predominantly the limbic component of dopamine recipient forebrain regions.

## 4. Discussion

The hypothesis positing that ASD may be linked to either functional impairment [1] or defective developmental processes [4,5,50] within the DAergic system has been proposed previously. Circuits of the striatum, which rely substantially on DAergic input, have been conceptualized as pivotal elements in the pathogenesis of various ASD phenotypes (for review, see Fuccillo [6]). According to earlier reports, the social deficits observed in VPA-exposed rats may be attributed to a developmentally regulated dysfunction of the DAergic system [47], suggesting that DAergic hypoactivity may also explain certain ASD symptoms [9]. Furthermore, the high rate of comorbidity between ASD and affective disorders, such as bipolar disorder, anxiety, and depression [51], provides additional support for the notion that certain forms of autism are associated with DAergic system dysregulation. Evidence linking the DAergic system to the genetic underpinnings of human ASD includes the involvement of a de novo missense mutation in the dopamine transporter gene [1] and alterations in the dopamine-3-receptor gene (DRD3) [2], for an overview, see Nguyen et al. [3].

Dysfunction within the DAergic system has also been verified in various animal models of autism [7,8,52,53]. This includes alterations in DA and DA receptors mediating VPA-related behavioral changes in rats [14,52,53], changes in methamphetamine-evoked (DA-dependent) hyperlocomotion in mice [11], and modified distribution of mesencephalic DAergic neurons in domestic chicks [41]. Our research groups were among the first to demonstrate distinct and cohesive neuroanatomical and neurochemical responses in the murine DAergic system [42].

The suggestion that DAergic innervation may influence the organization of striatal development has been known for some time [15]. Subsequently, the developmental sequence of DAergic centers and pathways was thoroughly described, particularly in rats. By E17, a prominent bundling of DA neurites occurs [15]. In the striatal targets, the DA fibers enter first as distinct “patches” or “dopaminergic islands” at birth, to get gradually dispersed by the end of postnatal week 3. Lack of persistence and growth of these islands were found to be characteristic features of the weaver mutant model [54]. Previous studies have described the pre- and postnatal development of DAergic neurons of the ventrotegmental–accumbens (mesolimbic) pathway under the effect of chemorepellent or chemoattractant factors, specifically involved in the segregation of distinct dopaminergic pathways/connections [4].

The prolonged growth of DA + axons, forming symmetrical axospinous synapses [48], indicates that the DAergic system may regulate pattern formation of the entire striatum and NAc. Generally, the striatum takes a long time to achieve an adult-like pattern of neuropeptide expression, also requiring synchronous and compartment-selective afferent innervation [55]. A derangement of nigrostriatal and ventral tegmentostriatal axons has been reported to precede neurodegeneration in PET studies in human subjects suffering from Parkinson’s disease [56].

### 4.1. Dopaminergic System and Ventrobasal Forebrain Anatomy, Relevance to ASD

Dopaminergic projections originating from the midbrain differentially innervate the forebrain. The dorsolateral striatum, or CPu, is predominantly innervated by the SNc, whereas the ventrobasal forebrain, which includes the NAc and OT, primarily receives its input from the VTA [57,58]. The mesolimbic DAergic reward system overlaps anatomically and functionally with the Social Behavior Network (SBN) [59], assembling into the social decision-making network [60,61]. The latter is substantially involved in decision-making, reward assessment, and social attraction [62]. This central role is based on its structural composition and extensive connections with limbic forebrain centers, such as the ventral pallidum, olfactory tubercle, extended amygdala, septum, bed nucleus of the stria terminalis, prefrontal cortex, and specific hypothalamic nuclei. These functions are often implicated as potential domains of impairment in ASD [62,63]. Pattern formation and wiring of the ventrobasal forebrain is thought to be tightly regulated by DAergic input [16].

A critical point in the etiology of ASD may be insufficiency or retardation of this DA-regulated developmental process, specifically by missing the critical time window during embryonic development This failure to properly establish or mature the DAergic circuitry could result in a subsequent weaker or inadequate response to social signals in later ages. Depending on the efficacy of compensatory mechanisms implemented by the developing brain, this initial developmental defect may persist into adulthood, ultimately manifesting as the various forms of ASD.

### 4.2. Synaptic Plasticity and Dopaminergic Development in VPA Models

Prior research has established that synaptic reorganization is a significant feature in the limbic forebrain following prenatal valproic acid (VPA) exposure. One such alteration is enhanced dendritic arborization and spinogenesis in the NAc, which may indicate defective synaptic pruning [64].

Conversely, reduced spine density and lower expression of synaptic protein mRNAs was observed in the hippocampus of VPA-exposed mice [65].

### 4.3. Linking Defective Dopaminergic Input to Limbic Forebrain Changes in VPA Models

Accumulating evidence strongly supports the notion of an attenuated dopaminergic input to the limbic forebrain at P7.

Defasciculation: A clear defasciculation (disruption of fiber bundling) of TH+ fibers in the mesolimbic tract is evident in VPA-exposed mouse pups at P7 [42]. Similar mesolimbic pathway alterations have been reported in children with autism [43]. This defasciculation may result in a net loss of DAergic fibers reaching limbic forebrain targets, and misdirection of tegmental DAergic fibers from the ventral (limbic) to the dorsal striatum.

Reduced DA and TH protein: A decrease in DA levels was measured by ELISA in the Nucleus Accumbens (NAc), but not the Caudate-Putamen (CPu) (though the Olfactory Tubercle (OT) may have disproportionately contributed to the NAc measurement) [42]. A decrease in the synaptic component of the TH protein (assessed by immunoblotting) in the NAc (including OT) [44] and a diminished incidence of TH+/CR+ or TH+/CB+ juxtapositions in the NAc and OT, respectively [45], both support a genuine attenuation of DAergic input during this critical early postembryonic phase. Despite the observed reduction of total CB protein in the OT of VPA-exposed animals, the number of CB+ perikarya remained unchanged, suggesting that the decrease in total CB reflected a reduction in its expression within the neuronal processes. This reduction may decrease the available synaptic surface on CB+ neurons, thereby further reducing the probability of forming or maintaining synaptic contacts with TH+ afferent axons.

Reduced mean voxel intensity of TH immunolabel in the OT and NAc (but not in CPu) in whole mount tissue cleared (iDISCO) brain specimens of VPA exposed mice (current study). One obvious advantage of this method is that it was able to find significant decrease of TH not only in OT, corroborating the robust drop of TH (like our previous report obtained by section IC and confocal microscopy) but also in the NAc (not detected by the previous morphometric method).

### 4.4. Compensatory Mechanisms and Vulnerability

While some literature sources did not confirm a reduction of TH+ cells in the VTA or a decrease in TH protein in the NAc at a later time point (P21) [46], other reports point to an early deprivation of DA, followed by restoration. Upregulation of DA receptors in the NAc of mice prenatally exposed to VPA [47] likely represents a compensatory mechanism following an earlier ligand (DA) deprivation (as detected at P7). According to Kudrin et al. [66,67] prenatal exposure to VPA caused a decrease in all parameters of monoaminergic neurotransmission in the striatum (but not in other investigated brain structures) of P15 mouse offspring, followed by restoration to the corresponding values of control group by P64. Studies on mutant mice (En1cre/+; Otx2^flox/flox^) with reduced midbrain DAergic neurons showed selective TH depletion in the ventrobasal forebrain (OT and NAc shell), but not the dorsal striatum, alongside an upregulation of D1 and D2 receptors and phosphorylated DARPP-32 [68].

This evidence suggests that the DA recipient ventrobasal forebrain is intrinsically highly vulnerable to defects, whether induced by environmental (VPA) or by genomic/transcriptional factors, that disrupt axonal pathfinding and neural organization. Previous studies, supported by current findings, suggest that pattern formation in the OT plays a prominent role in early DAergic regulation (for review [69]). Such reorganization, particularly in motivation-associated forebrain regions such as OT, has been observed in non-human primates [55], and it may underlie certain behavioral deficits characteristic of human ASD.

### 4.5. Translational Significance of Findings

Our findings on DAergic pathway reduction in VPA exposed mice align with an fMRI study reporting modified connectivity within the mesolimbic reward pathway (from ventral tegmental area to the NAc and other ventrobasal forbrain target regions) in human ASD subjects [43]. Based on simultaneous [^11^C] raclopride PET and fMRI imaging investigation of striatal DA binding, the ASD cohort demonstrated decreased phasic dopamine release to incentives in the bilateral putamen and left caudate, as well as increased functional connectivity between a PET-derived right putamen seed and the precuneus and insula [70]. An increasing number of imaging studies, aimed to find early diagnostic markers for ASD, will likely be correlated to structural and functional alterations of DAergic pathways.

## Data Availability

The original data presented in the study are openly available at: https://drive.google.com/file/d/16-EXu6lQJJ24gVvX2VLQ75FELolPngu0/view?usp=drive_link, access on 4 March 2026.

## Supplementary Materials

The following supporting information can be downloaded at: https://www.mdpi.com/article/10.3390/biomedicines14030590/s1, Video S1: Representative video sequence depicting a light sheet microscope image stack from a TH immunostained (red) and tissue-cleared brain specimen of P7 control mouse, loaded onto Imaris 9.9.1. for quantitative analysis. The color-coded regions of interest (ROI) marked out in 3D represent the following forebrain regions: nucleus accumbens (blue), tuberculum olfactorium (yellow). Video S2: Representative video sequence depicting a light sheet microscope image stack from a TH immunostained (red) and tissue-cleared brain specimen of P7 VPA-exposed mouse, loaded onto Imaris 9.9.1. for quantitative analysis. The color-coded regions of interest (ROI) marked out in 3D represent the following forebrain regions: nucleus accumbens (blue), tuberculum olfactorium (yellow).
